# Nurses’ Clinical Reasoning Process: A Grounded Theory Study

**DOI:** 10.3390/healthcare14020230

**Published:** 2026-01-16

**Authors:** Susana Mendonça

**Affiliations:** 1 School of Nursing of Lisbon, University of Lisbon, 1649-004 Lisboa, Portugal; susana.mendonca@esel.pt; Tel.: +351-966455933; 2 Comprehensive Health Research Center (CHRC), 7004-516 Évora, Portugal; 3 Center of Innovative Care and Health Technology (CiTchCare), 2414-016 Leiria, Portugal

**Keywords:** clinical reasoning, diagnostic nursing assessment, grounded theory, nurses, patient, therapeutic intervention

## Abstract

**Highlights:**

This study examines the clinical reasoning process of nurses working in emergency departments. The resulting Nursing Clinical Reasoning Model highlights the dynamic, contextual, and relational nature of decision making in emergency care.

**What are the main findings?**

- The Nursing Clinical Reasoning Model conceptualizes clinical reasoning as a dynamic and iterative process.
- Clinical reasoning is embedded in the nurse–patient–family relationship and is shaped by contextual factors that influence how nurses assess, intervene, and respond to patient needs.

**What is the implication of the main finding?**

- Applying this model in emergency settings may lead to an earlier recognition of patient severity, faster and more consistent interventions, and improved patient safety. It also implies benefits for training and protocol development by clarifying the subprocesses that underpin nurses’ decision making.

**Abstract:**

**Background**: Nurses’ clinical reasoning is increasingly being recognized as a core competence that enhances the quality and safety of care across diverse healthcare settings. Nurses with refined clinical reasoning skills contribute significantly to improved health outcomes and broader health gains. In emergency departments, this competence is essential to rapidly assessing complex problems, anticipating complications, and preventing the deterioration of patients’ clinical conditions. Such expertise enables nurses to discern the severity of clinical situations quickly and intervene effectively. **Objectives**: The aims of this study were to analyze the clinical reasoning process of nurses and develop a theory that explains this process in emergency care settings. **Methodology**: This qualitative study explored the following research question: “How do nurses enact the clinical reasoning process in emergency departments?” The Grounded Theory methodology was used, with a theoretical sample of 20 nurses. Data collection methods included in-depth interviews, participant observation, and field notes. **Results**: The theoretical analysis identified clinical reasoning as a substantive theory composed of two subprocesses: Diagnostic Nursing Assessment and Therapeutic Nursing Intervention. Nurses’ clinical reasoning determines two levels of patient severity—Level I, life-threatening situations (immediate risk), and Level II, non-life-threatening situations (expressed problems)—according to which nursing interventions are adjusted. **Conclusions**: The Nursing Clinical Reasoning Model is a dynamic and continuous process that involves both Diagnostic Nursing Assessment and Nursing Therapeutic Intervention. It is deeply rooted in the nurse–patient–family relationship and is shaped by the specific care context, which influences nurses’ assessments and interventions and patients’ responses and behaviors.

## 1. Introduction

### Clinical Reasoning Among Nurses: Conceptual Background

Reasoning is a characteristic that distinguishes humans from other living beings, encompassing the ability to think, understand, analyze, and make decisions based on information and experience. It is a personal, conscious, and active process supported by logical mechanisms that enable individuals to interpret data and identify solutions to problems [1,2].

Clinical reasoning is a complex cognitive process essential to the assessment and management of individuals’ health status. In emergency care settings, this competence is crucial for nurses to make sound and well-founded clinical decisions, thereby ensuring the quality and safety of care.

Among nurses, clinical reasoning plays a vital role in assessing patients’ clinical conditions, identifying health problems, and making rapid and accurate decisions about appropriate interventions. Ensuring safety in clinical assessment, problem identification, and sound decision making constitutes a core professional responsibility that requires education and continuous training.

Working in emergency departments—where patients are often in critical condition and clinical circumstances can change rapidly—places considerable pressure on nurses. These professionals must make rapid, evidence-informed decisions in high-risk situations [3,4]. Such a dynamic environment increases the risk of burnout and heightens vulnerability in decision-making processes. Interventions often require immediate responses, leaving little time for reflection. Clinical reasoning is, therefore, recognized as a key criterion of nursing competence [5,6,7]. Effective reasoning allows nurses to anticipate complications, foresee potential problems, and act proactively in critical situations.

The reasoning process integrates assessment, diagnosis, decision making, intervention, and evaluation. It lies at the core of nursing competence, supporting accurate diagnoses and well-founded clinical judgments [4,6,8]. To ensure safe and effective care, nurses must analyze diverse information and draw on their experience to address the complex challenges of clinical practice. This process fosters patient safety and leads to better health outcomes.

Several authors [6,9] describe clinical reasoning as the ability to guide actions based on knowledge and experience, materializing through problem solving, decision making, planning, and justification of interventions. Clinical experience, critical thinking, a solid knowledge base, and the integration of evidence-based practice are among the essential competencies that support decision-making processes [4,10]. Furthermore, nurses’ proximity to patients and the relationships they establish enhance their ability to assess situations and adjust decisions accordingly [5,8,11].

In healthcare delivery, it is crucial that nurses possess strong clinical decision-making skills and are valued and recognized for this responsibility. Nursing work has become increasingly complex in the face of rapid technological and scientific advances, introducing new challenges and responsibilities that justify the profession’s recognition within healthcare institutions and society.

There has been growing investment in nurse education and research on clinical reasoning—both to develop theoretical models and to improve clinical practice. Educational strategies such as clinical simulation have proven effective in strengthening this essential competence [12,13,14].

From a research perspective, clinical reasoning has been examined through both deductive and inductive approaches, providing a comprehensive understanding of the phenomenon. Patricia Benner, a seminal figure in this field, developed a line of research that theorizes how nurses make decisions and how this ability evolves through professional experience [6,7]. She pioneered the concept of clinical judgment, understood as the capacity to make deliberate, competent, and contextually grounded decisions [6,9].

However, significant challenges remain in deepening the understanding of clinical reasoning to improve education, training, and clinical practice. The ongoing exploration of this process is essential to minimizing errors and adverse events in healthcare—issues particularly relevant among newly graduated nurses [5,15,16,17].

Studies by Aiken found that nurses with higher academic qualifications achieve better patient outcomes [17]. Additionally, an analysis of healthcare incidents by the New South Wales public health system [18] identified three main causes of adverse outcomes: diagnostic errors, failures in treatment implementation, and the inadequate management of complications. These findings reinforce the need to invest in research on clinical reasoning, particularly in emergency nursing, where timely and accurate decision making is critical to patient safety.

Accordingly, this study seeks to explore the clinical reasoning process of nurses working in emergency departments, contributing to the advancement of knowledge and the improvement of nursing care practices.

## 2. Materials and Methods

### 2.1. Theoretical Framework

Grounded Theory (GT) was adopted as the theoretical and methodological framework of this study because it enables an in-depth exploration of social processes as they unfold in real-world contexts. In this study, GT provided a robust framework for understanding the clinical reasoning process of nurses working in emergency care settings.

This method is grounded in the perspective of symbolic interactionism, which views individuals as actors engaged in a continuous process of problem solving, where meanings are constructed, negotiated, and transformed through social interactions. From this perspective, clinical reasoning is understood as a dynamic, situated, and socially mediated process, making this approach particularly coherent with the objectives of the present study.

A distinctive feature of GT lies in its dual capacity to provide the meaning, understanding, and description of the phenomenon under study while simultaneously facilitating the generation of theory [19]. According to Charmaz, GT is particularly advantageous because it systematically focuses on process analysis, allowing empirical observations to be interpreted and integrated into a theoretical explanation grounded in participant’s experiences [19].

Within this constructivist perspective, data collection and analysis occur in an iterative and interconnected manner, enabling the progressive refinement of concepts and categories as the theory develops.

Consistent with this perspective, this study adopted a qualitative, inductive approach based on Charmaz’s constructivist Grounded Theory. As an inductive method, GT allows theory to be generated from empirical data, revealing processes and meanings that are not immediately observable.

We situated data collection and analysis within the natural context of emergency care practice to enable theoretical explanations to emerge from nurses’ direct interactions and everyday clinical work.

This framework proved particularly appropriate for capturing the complexity of nurses’ clinical reasoning, supporting the construction of concepts, categories, and subcategories that reflect the multifaceted and context-dependent nature of the phenomenon under investigation.

### 2.2. Study Design

This qualitative study employed Grounded Theory (GT) procedures for data analysis, ensuring a systematic and rigorous approach.

GT was operationalized through iterative cycles of data collection and analysis, in which emerging insights informed subsequent sampling and analytical decisions.

The analytical process followed successive and interconnected stages of coding, including initial and focused coding, supported by analytic memo writing. Memos were used systematically to document analytical decisions, explore relationships among categories, and support theoretical integration; this iterative process enabled progressive abstraction and conceptual development. Analytical stages unfolded through successive and overlapping phases, with theoretical saturation being achieved through multiple analytical cycles that evolved in a spiral and progressively complex manner.

Multiple data collection methods—semi-structured interviews, participant observation, and field notes—were used to enable triangulation. This approach enhanced analytical depth and credibility by enabling a comparison across different perspectives and the identification of convergences, discrepancies, and contradictions across different data sources, in accordance with Grounded Theory principles [20].

Data collection and analysis were conducted within the natural context of emergency care practice, which allowed theoretical explanations to emerge from nurses’ everyday clinical activities. This contextualized approach supported the development of a grounded and empirically anchored explanation of the clinical reasoning process.

#### Quality Criteria and Trustworthiness

This study was conducted and reported in accordance with the Consolidated Criteria for Reporting Qualitative Research (COREQ), and specific strategies were adopted to ensure credibility, dependability, confirmability, and transparency throughout the research process.

Credibility was enhanced through prolonged engagement in the field, the triangulation of data sources (semi-structured interviews, participant observation, and field notes), and constant comparison during data analysis. The iterative nature of data collection and analysis allowed emerging categories to be continuously refined and verified against new data. In addition, analytic memos were used to document evolving interpretations and to support reflexive engagement with the data.

Dependability was ensured through a systematic and well-documented analytical process. Coding decisions, category development, and theoretical integration were recorded in the analytic memos, creating an audit trail that allowed the progression of analysis to be traced. The use of iterative analytical cycles and constant comparison further contributed to the consistency and stability of the findings.

Confirmability was addressed through reflexive practices and analytical transparency. Reflexive and analytic memos were used to document assumptions, decision-making processes, and shifts in interpretation. Regular discussions with doctoral supervisors provided external analytical scrutiny, supporting the examination of alternative interpretations and minimizing the influence of researcher bias.

Transparency and rigor were further strengthened by explicitly linking analytical decisions to empirical data and by reporting this study in accordance with the COREQ guidelines, which allows readers to assess the methodological integrity and trustworthiness of the findings.

### 2.3. Participants and Study Setting

Participant selection followed specific criteria for defining clinical expertise. Nurses were required to have a minimum of five years of professional experience in the emergency department, perform duties across all sectors of the service, and demonstrate availability and motivation for voluntary participation, consistently with recommendations in the literature for identifying expert practitioners. Participants were recruited through purposive sampling: the nurse manager of the emergency department disseminated an invitation to all nurses in the service, and those who met the inclusion criteria and expressed willingness to participate were subsequently contacted by the research team. Exclusion criteria included nurses with less than five years of emergency experience or temporary nurses, and those who were unavailable or unwilling to participate. Recruitment continued until theoretical saturation was achieved.

A total of 20 nurses participated, all of whom volunteered and provided informed consent after receiving detailed information about this study’s objectives and procedures. All participants were engaged in direct patient care within the emergency department. Some also carried additional responsibilities during their shifts, such as auditing clinical procedures and coordinating or managing nursing teams.

The present study was conducted over a six-month period in a central, multidisciplinary public hospital located in the region of Lisbon, Portugal. Data collection occurred in a high-volume emergency department that provides care to a large number of patients daily, with an average of approximately 550 users per day. This environment exposes nurses to a wide spectrum of clinical presentations, from critically ill to non-urgent cases, and offers rich opportunities to observe clinical reasoning in real time within a dynamic, complex, and rapidly changing setting.

To ensure the comprehensive observation of participants’ reasoning processes, we observed each nurse during four eight-hour shifts covering all emergency department sectors—triage, resuscitation rooms, ambulatory/balcony areas, and observation rooms. The researcher acted as a participant observer, remaining in close proximity to clinical practice while adopting measures to minimize bias, including systematic field note recording, reflective memo writing, and maintaining analytical distance during data interpretation.

### 2.4. Sampling and Theoretical Saturation

Theoretical sampling was conducted concurrently with the data collection process and analysis, in accordance with the principles of constructivist Grounded Theory. Initial interviews informed subsequent sampling decisions, which were guided by the need to elaborate, refine, and contrast emerging categories rather than to achieve numerical representativeness.

As the analysis progressed, additional participants were purposefully selected to explore emerging categories in greater depth and examine variations in clinical reasoning across different situations. Sampling decisions were, therefore, driven by analytic needs, with the aim of clarifying properties, dimensions, and relationships within and among categories.

Theoretical saturation was considered to be achieved when successive interviews no longer generated new conceptual insights, properties, or dimensions relevant to the core categories. In this study, the saturation of the categories and subcategories related to nursing diagnostic assessment, clinical reasoning, and the therapeutic nursing relationship began to be observed from the eighth interview onward. At this stage, data analysis revealed repetition and stability in the identified patterns, with no emergence of new conceptual elements.

The first category to reach saturation was nursing diagnosis, followed closely by clinical reasoning. Subsequently, the category related to the therapeutic relationship with patients and families was further developed and refined until no additional analytical properties emerged. This progression reflected the centrality of these dimensions in Nursing Clinical Reasoning Processes within emergency care settings.

Saturation was further supported by:

- Constantly comparing data across interviews;
- Examining whether new data added analytical depth or altered existing interpretations;
- Analyzing deviant or contrasting cases to test the robustness of emerging categories.

Participant observations and analytic memos played a central role in clarifying and refining the understanding of nurses’ interactions with patients, families, and other healthcare professionals. These materials supported the visualization of relational dynamics and decision-making processes, strengthening the analytical depth and theoretical coherence of the emerging model.

### 2.5. Data Collection Techniques

Data collection (interviews, observations, and field notes) was conducted by a single researcher to ensure consistency in the procedures, while data analysis was carried out collaboratively. The primary researcher performed the initial coding, and the two senior researchers—who acted as supervisors of this study—reviewed and discussed the emerging categories and theoretical links in regular analytical meetings. Any differences in interpretation were resolved through discussion until consensus was achieved. This collaborative process enhanced the rigor and trustworthiness of the analysis. The interviews were audio-recorded and transcribed using Microsoft Word (Microsoft Corporation, Redmond, WA, USA). The transcribed data were subsequently coded and analyzed using NVivo (v10; QSR International Pty Ltd., Melbourne, VIC, Australia).

#### 2.5.1. In-Depth Interviews

The in-depth interview method [20,21,22] was selected as it is well suited to exploring participants’ behaviors, beliefs, experiences, interactions, and ideas within the context of their everyday professional practice. This technique enabled an understanding of the phenomenon through the participants’ own language and perspectives, valuing their narratives and lived experience [23,24]. All interviews were conducted by a single researcher and took place in carefully selected settings—quiet, isolated rooms free from interruptions—to ensure a safe, calm, and comfortable environment that fostered participants’ trust, focus, and freedom of expression. We used a semi-structured interview guide consisting of open-ended questions designed to encourage reflection and detailed descriptions of experiences such as the following:

- “Recall a recent clinical case and describe the events in detail.”
- “What did you think?”
- “How did you think?”
- “What decisions did you make and how?”
- “What was the most challenging aspect?”

Data analysis was carried out continuously, comparatively, and iteratively, involving a back-and-forth process between data collection and interpretation until theoretical saturation was reached. This dynamic approach allowed for a deeper understanding of the phenomenon and ensured that all relevant aspects were incorporated into the construction of the emerging theory, in line with the principles of constructivist Grounded Theory [19,25].

#### 2.5.2. Participant Observation, Field Notes, and Memos

In addition to interviews, participant observation and field notes, complemented by analytical memos, were employed to enrich and validate the data collected. This combination ensured methodological triangulation and strengthened the overall consistency and credibility of the research process.

Participant observation [26,27] focused on two main dimensions:

(I) General observation of clinical reasoning: The researcher simultaneously observed several nurses working across different sectors of the emergency department, recording nurse–patient interactions and focusing on clinical reasoning processes as they unfolded in real time.
(II) Individualized observation: At specific points, the researcher followed a single nurse during care delivery, documenting both observable actions and the underlying reasoning processes—such as questions asked, the use of technological tools for assessment, and the consultation of clinical records.

These observations were conducted during four structured eight-hour shifts for each participant, ensuring consistency and allowing for a comparison across different clinical contexts within the emergency department.

Field notes [26,27] were used to record behaviors, interactions, and emotional responses during clinical encounters, serving as detailed descriptive records of the observed context. In parallel, analytical memos [19] played a crucial role in interpreting observations, refining emerging categories, and validating data derived from both interviews and observations. Together, these documents captured behavioral and emotional nuances, deepened the understanding of the phenomenon, and contributed to a robust triangulation of the data sources.

### 2.6. Data Analysis Procedures

Data analysis was conducted concurrently with data collection, following the constant comparative method central to Grounded Theory. Each interview was transcribed verbatim and analyzed immediately after completion. Initial coding involved the line-by-line examination of the data to identify key actions, processes, and meanings. These preliminary codes were then compared across interviews to reveal similarities, differences, and emerging patterns. Focused coding was used to synthesize the most significant and frequent codes into conceptual categories that best represented the participants’ experiences and perspectives.

Memo writing was used throughout the analytic process to capture insights, theoretical reflections, and connections among categories, facilitating the refinement of concepts and their properties. As analysis progressed, relationships among categories were explored to build a coherent and integrative theoretical framework explaining the clinical reasoning process of nurses in emergency care.

The constant comparison of data, codes, and categories ensured that the emerging theory remained grounded in participants’ accounts while allowing for increasing levels of abstraction.

Measures to reduce potential bias arising from the participant-observer role were consistently implemented and includes the triangulation of interviews, observations, field notes, and memos; the systematic cross-checking of interpretations with new data; and ongoing reflexive documentation to ensure analytical transparency.

The analysis continued until all categories were theoretically saturated and integrated into a final conceptual model explaining nurses’ clinical reasoning in emergency care.

All data were collected in Portuguese, the participants’ native language, to ensure natural expression and richness of meaning. Interviews were transcribed verbatim and analyzed in the original language to preserve semantic, contextual, and cultural nuances throughout the analytic process.

Translation into English was performed exclusively for the purpose of reporting selected excerpts in this manuscript. To maintain meaning equivalence and analytic validity, translations were conducted by the research team, all fluent in both languages, with careful attention to conceptual accuracy rather than literal correspondence. When necessary, translations were discussed among researchers to ensure consistency with the original meaning of the participants’ statements.

### 2.7. Reflexivity and Management of Researcher Bias

Given the interpretative nature of constructivist Grounded Theory, particular attention was paid to reflexivity and the management of potential researcher bias throughout all stages of this study.

The researcher maintained a reflexive stance from data collection to analysis, acknowledging prior professional experience in emergency care and its potential influence on data interpretation. To address this, reflexive memos were systematically written throughout the research process to document assumptions, emerging interpretations, analytical decisions, and moments of uncertainty. These memos served as an audit trail and supported critical self-reflection regarding how meanings were being constructed.

During data analysis, analytic memos were used not only to support category development but also to question preliminary interpretations, explore alternative explanations, and document shifts in analytical thinking. This process allowed emerging categories to be continuously challenged and refined, rather than accepted at face value.

Participant observation and interviews were analyzed in parallel, enabling a comparison between reported practices and observed behaviors. This triangulation supported the reflexive examination of potential observer effects and contributed to a more nuanced understanding of clinical reasoning in practice.

Interpretative decisions were further examined through ongoing dialog with doctoral supervisors, who provided critical feedback and encouraged the exploration of alternative analytical perspectives. This process contributed to minimizing individual bias and strengthening the credibility and coherence of the emerging theoretical model.

Through these reflexive practices, this study sought to ensure transparency, analytical rigor, and methodological integrity, in line with the principles of constructivist Grounded Theory.

### 2.8. Ethical Considerations

Ethical approval for this study was obtained from both the University Ethics Committee and the Hospital Ethics Committee (CH/CE/10-02-2018). All participants were fully informed about the purpose of this study, the researcher’s role, and the conceptual background before data collection began. Written informed consent was obtained from all participants, who were reminded that their participation was voluntary and that they could withdraw at any stage without consequence.

Data were stored electronically on a password-protected computer, accessible only to the research team. Ethical considerations included obtaining informed consent and ensuring the confidentiality of all data collected. Although full anonymity during observation could not be guaranteed, participants were informed that all interviews, observations, and field notes would be fully coded and anonymized during transcription, analysis, and reporting, ensuring that no individual could be identified in the final study. Participants were also given the option to receive a summary of the study findings upon completion.

## 3. Results

### 3.1. Participant Characteristics

A total of twenty nurses participated in this study. Participants’ ages ranged from 28 to 48 years, with a mean age of 36.8 years (SD = 5.72). The sample included eleven females and nine males, reflecting a relatively balanced gender distribution.

Participants had an average of 14.9 years of professional experience (SD = 5.37), of which 11.3 years (SD = 5.46) were spent working specifically in emergency care. This indicates that most participants possessed substantial clinical and contextual experience, allowing them to articulate complex insights into their clinical reasoning processes.

Regarding educational background, ten participants held a Bachelor of Science in Nursing (BSN) and ten held a Master of Science in Nursing (MSN), demonstrating a heterogeneous group in terms of academic qualification. This diversity in both professional and educational experience enriched the analysis, enabling comparisons between different levels of expertise and reflective depth within the sample.

Following the characterization of participants, the qualitative analysis of the collected data is presented. The interpretation followed the principles of constructivist Grounded Theory, allowing categories and subcategories to emerge progressively through the constant comparison of data. This analytical process enabled an in-depth understanding of how nurses construct their clinical reasoning in emergency care settings, revealing meanings, patterns, and variations in their practices and decision-making processes.

### 3.2. Clinical Reasoning—Central Category

The data analysis revealed the emergence of the central category: Clinical Reasoning. This term was frequently mentioned by participants and became a core concept that guided the analytical process. In the interviews, twelve nurses explicitly referred to the term clinical reasoning in their narratives. In the remaining interviews, participants used alternative expressions such as clinical judgment, critical thinking, and clinical eye to describe similar cognitive processes. These nuances can be observed in the following excerpts:

“Clinical reasoning in the emergency department is very important. It stems from our theoretical knowledge combined with our ability to observe the patient, to listen to what the patient is telling us, to what the family members are saying—everything we are able to gather. Because we are much closer to the patient, we can access information more quickly, which helps us in establishing a diagnosis.”

(Participant5_CA_0309)

“Clinical reasoning is a set of experiences, knowledge, and understanding that we acquire over time through our practice.”

(Participant2_ROI_0218)

The use of different terms to describe the same phenomenon reveals variations in how nurses construct and interpret their clinical reasoning processes. This linguistic plurality reflects different levels of awareness and conceptualization of professional thinking, as illustrated in the following excerpts:

“The clinical eye is looking at the person without looking at the monitor… looking directly at the patient. There’s something that tells us—several facial signs: if the person is agitated, confused, the color of their face. There are many signals that draw our attention and that we know how to identify; we know this patient is not well, and we need to look deeper. It’s often in this way that we manage to prioritize care, anticipate complications—I think it’s a key foundation for preventing problems, managing priorities, and anticipating complications.”

(Participant9_PA_0313)

The analysis of the narratives revealed that although the terminology varied, all participants referred to the same conceptual core—a process of thinking and acting intentionally and knowledgeably in the face of complex clinical situations. Regardless of the term used—clinical reasoning, clinical judgment, critical thinking, or clinical eye—the participants described a cognitive path involving observation, the interpretation of signs, the anticipation of complications, and decision making.

This terminological diversity reflects not only personal styles of expression but also different levels of experience and professional background. Thus, clinical reasoning is understood as a complex cognitive process that involves collecting, analyzing, and interpreting information, leading to decision making and culminating in nursing interventions aimed at addressing or resolving the patient’s problem. This process begins at the first contact between nurse and patient, forming the foundation for assessment and subsequent care planning.

In these excerpts, the different ways of conceptualizing clinical reasoning become evident, revealing more specific aspects of how meanings are constructed and negotiated in everyday emergency care practice. From this central concept, two interrelated subcategories emerged: Diagnostic Nursing Assessment and Therapeutic Nursing Intervention.

#### 3.2.1. Subcategories of the Nursing Clinical Reasoning Process in Emergency-Context Diagnostic Assessment and Therapeutic Nursing Intervention

In the interview excerpts analyzed, different ways of conceptualizing the Nursing Clinical Reasoning Process became evident, revealing specific aspects of how meanings are constructed, interpreted, and negotiated in daily practice within emergency settings. From this central concept, two interrelated subcategories emerged—Diagnostic Nursing Assessment and Therapeutic Nursing Intervention—which, although distinct, develop in an interdependent and continuous manner, representing two complementary dimensions of the same cognitive, technical, and relational process.

##### Subcategory 1: Diagnostic Nursing Assessment

Diagnostic Nursing Assessment constitutes the first stage of the Nursing Clinical Reasoning Process and represents the starting point for all subsequent decisions in the emergency context. It is at this moment that the nurse rapidly and knowledgeably interprets the patient’s signs and symptoms, transforming observable data into preliminary clinical judgments that guide immediate action.

**Figure 1:**
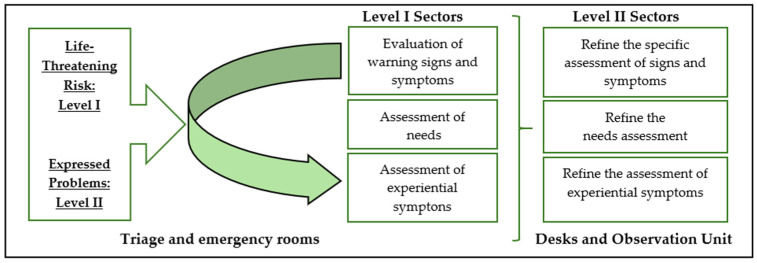
Diagnostic Nursing Assessment process.

From the analytical process, two central conceptual properties emerged:

- Life-Threatening Risk (Level I): The prioritization of immediate interventions for patients in critical condition, i.e., a situation in which a rapid response is crucial to survival.
- Expressed Problems (Level II): The identification of and response to complaints or needs explicitly reported by the patient, even when they do not represent an immediate life-threatening situation (Figure 1).

Diagnostic Nursing Assessment is characterized by its systematic, dynamic, and continuous nature, sustained by clinical observation, active listening, and the integration of information from various sources: the patient, the family, and the prehospital team [15]. The nurse simultaneously mobilizes scientific knowledge, experience, and clinical intuition—elements widely recognized in the literature as essential to effective clinical reasoning [4,9,28].

Key elements of this process include structured data collection, rapid prioritization of needs, and the constant monitoring of the patient’s clinical progress. The nurse’s reasoning is continuously updated as new information becomes available, forming a perception–interpretation–reassessment cycle that ensures the appropriateness of interventions to the patient’s clinical condition.

During this process, nurses frequently rely on guiding questions that structure clinical thinking and facilitate immediate decision making:

“Is the patient breathing?”
“Is there a pulse?”
“Can the patient speak?”
“What are the signs and symptoms?”
“Are there any pre-existing conditions?”
“Are there visible or hidden injuries?”
“What are the patient’s limitations?”
“What are the patient’s emotional needs or concerns?”
“What is the role and support of the family?”
“What resources are available?”

One participant’s account highlights the methodical and technical character of this approach:

“Any patient I assess, I automatically use the ABCDE mnemonic (A—airway; B—breathing; C—circulation; D—disability/consciousness; E—exposure and visualization of all injuries), because it can be applied to a medical or surgical critical patient.”

(Participant2_ROI_20160218)

Another nurse emphasizes the importance of anticipating clinical changes, demonstrating the predictive reasoning that is essential in emergency settings:

“There are things that start to appear—you have to think about why these things are appearing, why there is this change in the patient, and many times they said: you have to anticipate situations, you have to realize that one situation can trigger another, and you have to be prepared. I think this is important for developing emergency nursing competencies.”

(Participant5_CA_0309)

Another participant also emphasized the diagnostic nursing value of family presence, noting that relatives often contribute essential information that refines the initial assessment:

“The presence of a family member is fundamental, because they can tell us what the patient was like before arriving here—what changed, how fast it changed, and what warning signs they noticed. Many times, it’s through the family that we understand what is really happening and can make a more accurate diagnostic assessment.”

(Participant17_AD_0412)

Beyond its role as a diagnostic source of clinical history, family presence was also described as contributing to the emotional stability of the patient and to the continuity of care beyond the emergency episode. One participant highlighted that although family members provide relevant information, their importance extends to reassurance, support, and post-discharge continuity:

“The family is very important in the emergency department because they are a valuable source of information, a source of safety for the patient, and they are the ones who will be with the patient after discharge.”

(Participant7_CR_0311)

Taken together, these accounts indicate that family involvement supports Diagnostic Nursing Assessment not only by supplying contextual clinical information, but also by contributing to patient safety, emotional support, and the continuity of care.

Contextual awareness and the ability to manage priorities also emerge as fundamental components of Diagnostic Nursing Assessment:

“First of all, it’s my duty to know what’s going on in the emergency department. What do I mean by that? I also have to know how many patients are waiting and the waiting time in each area. If I have a patient that I think should be seen immediately, I make that happen. Anything the patient mentions that doesn’t feel right, even if the vital signs are normal—afebrile, normotensive, normocardic, normoglycemic, normal oxygen saturation—I might still think the patient is about to decompensate.”

(Participant2_ROI_0218)

These accounts show that Diagnostic Nursing Assessment goes beyond the mere collection of objective data. It is an interpretive and anticipatory clinical act in which the nurse combines observation, judgment, and context, transforming immediate perceptions into reasoned decisions.

##### Subcategory 2: Therapeutic Nursing Intervention

**Figure 2:**
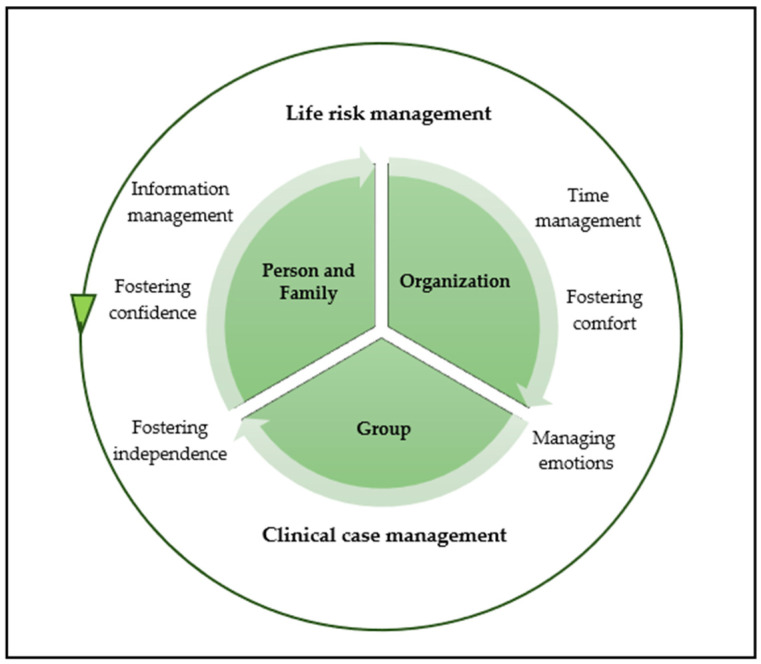
Multifocal character of Therapeutic Nursing Intervention.

Therapeutic Nursing Intervention represents the active phase of the Nursing Clinical Reasoning Process, during which the results of assessment are translated into concrete, focused, and intentional actions. Similarly to Diagnostic Nursing Assessment, this subcategory is based on two fundamental conceptual properties (Figure 2):

- Life-threatening risk (Level I).
- Patient-expressed problems (Level II).

Therapeutic Nursing Intervention is structured around multiple interdependent dimensions that reflect the complexity of care in emergency contexts:

1. The management of life-threatening risk, i.e., the continuous monitoring of vital functions and immediate intervention in response to any sign of instability.
2. Time management, i.e., the anticipation of complications and the adjustment of the timing of interventions according to the patient’s evolving needs.
3. Information management, i.e., clear and empathetic communication with the patient and family, fostering understanding and engagement in the therapeutic process.
4. Fostering comfort and trust, i.e., the creation of a safe and humanized environment, ensuring privacy, symptom control, and family support.
5. The management of emotions, i.e., helping the patient to rationalize and regulate emotions, reducing distress and facilitating adaptation to the health condition.
6. Fostering autonomy, i.e., encouraging health literacy and shared decision making, strengthening the patient’s independence during and after the emergency department stay.

The analyzed excerpts reinforce the relational and humanized nature of Nursing Therapeutic Intervention. Assertive and empathetic communication emerges as a key element in building trust and comfort:

“The relationship improves; that moment becomes more manageable when we put ourselves in the other person’s place, because we can easily understand their point of view and can explain things in the way we would like them explained to us. The person realizes that we are putting ourselves in their place and that we are making an effort to explain what they want to understand. In the end, it always comes down to being assertive.”

(Participant3_LI_0302)

Another participant highlights the value of personalized care:

“The relationship with the patient in the emergency department is established. I say this because sometimes a single sentence is enough—it’s enough for me to finish the triage and the patient realizes that I’m paying attention to them. When I finish triage, I explain that they will be referred to a particular specialty, and the patient feels that, feels that personalization, realizes they are not just another person who sat at the desk and is being triaged like everyone else.”

(Participant11_GA_0313)

Technical competence and professional confidence are also recognized by participants as pillars of effective intervention and therapeutic trust:

“Showing some confidence in what you’re doing automatically conveys security. Normally, I can only act with confidence and certainty when I know exactly what I’m doing. That’s why I mentioned competence. Showing confidence in what you do conveys trust and competence.”

(Participant3_LI_0302)

Therapeutic Nursing Intervention is, therefore, a multifocal and integrative process that combines scientific knowledge, technical skill, and human sensitivity. It requires the simultaneous coordination of cognitive and emotional dimensions, often under intense time and emotional pressure.

##### Synthesis

Together, Diagnostic Nursing Assessment and Therapeutic Nursing Intervention form a continuous, cyclical, and interdependent process in which clinical reasoning manifests as a spiral of perception, interpretation, decision, and action. This cycle ensures a nurse’s ability to act with safety, discernment, and empathy, even in contexts of high complexity and unpredictability.

The Nursing Clinical Reasoning Process, thus understood, constitutes the core of professional nursing competence, uniting science, technique, and humanity. This balance sustains the quality, safety, and humanization of care—fundamental pillars of nursing practice in emergency settings.

### 3.3. Conceptual Model of the Nursing Clinical Reasoning Process in Emergency Care

**Figure 3:**
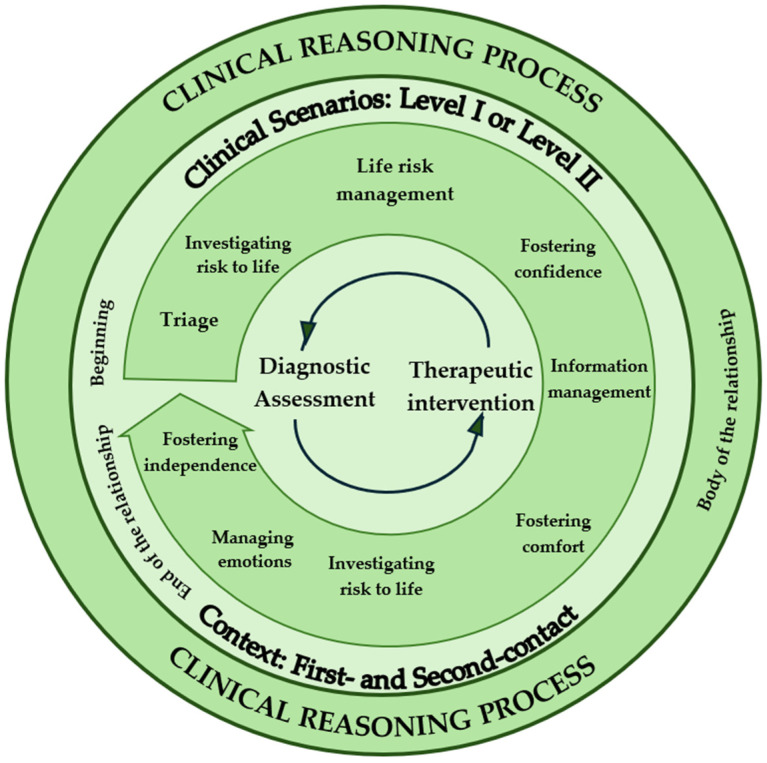
Clinical reasoning process of nurses working in the emergency department.

Figure 3 illustrates this conceptual synthesis, representing the Nursing Clinical Reasoning Process in emergency contexts as a dynamic, multidimensional, and context-dependent system. The model demonstrates how Diagnostic Nursing Assessment and Therapeutic Nursing Intervention are continuously interconnected within a spiral of perception, interpretation, decision, and action.

At the core of the model, the cyclical interaction between assessment and intervention symbolizes nurses’ ongoing process of reflection and adaptation in response to the patient’s clinical condition. Surrounding this core, the intermediate ring highlights the main operational axes that structure clinical reasoning in practice—life risk management, time and information management, emotional regulation, fostering comfort and confidence, and encouraging patient autonomy.

The outer ring represents the contextual and relational dimensions that influence clinical reasoning: the stages of the therapeutic relationship (beginning, body, and end) and the structural organization of the emergency department (first- and second-contact sectors). These layers show that clinical reasoning is not an isolated cognitive act but rather a situated, relational, and adaptive process, shaped by the interactions of the nurse, the patient, the latter’s family, and the clinical environment.

Overall, the figure visually integrates the findings of this study, depicting the Nursing Clinical Reasoning Process as a flexible and reflective mechanism that supports safe, humanized, and evidence-based care in emergency settings characterized by high pressure and complexity.

## 4. Discussion

The present study demonstrates that the Nursing Clinical Reasoning Process in emergency contexts is complex, dynamic, and multifaceted, reaffirming its centrality to clinical practice and patient safety [4,6,9,12,13,29]. Clinical reasoning emerges as a cognitive and relational process that integrates perception, interpretation, judgment, and action, continuously shaped by context and by the nurses’ individual characteristics [12,28].

The analysis revealed that clinical reasoning constitutes the central category of this study, expressed by participants through diverse terminology—clinical reasoning, clinical judgment, critical thinking, or clinical eye—yet all describing the same underlying cognitive process. This central category unfolds through two interdependent subcategories, Diagnostic Nursing Assessment and Therapeutic Nursing Intervention, which together form the core structure of nurses’ reasoning in emergency settings.

### 4.1. Alignment with the Diagnostic Nursing Assessment Subcategory

Consistent with the results, Diagnostic Nursing Assessment emerged as the initial and foundational moment of clinical reasoning. Participants described how they rapidly interpret signs and symptoms, differentiate between Level I life-threatening risks and Level II patient-expressed problems, and prioritize interventions accordingly.

This finding corroborates Tanner’s Clinical Judgment Model, which emphasizes perceptual sensitivity and pattern recognition as essential to accurate diagnostic judgments [9]. Similarly, recent studies in emergency nursing confirm that the rapid prioritization and anticipation of deterioration are critical determinants of patient outcomes [30,31].

The participants’ accounts also highlighted the cognitive strategies used in this diagnostic nursing phase—including ABCDE assessment (A—Airway; B—Breathing; C—Circulation; D—Disability; E—Exposure), contextual awareness, and predictive reasoning—mirroring the continuous perception–interpretation–reassessment cycle described in Section 3.

### 4.2. Alignment with the Therapeutic Nursing Intervention Subcategory

The second subcategory, Therapeutic Nursing Intervention, reflects the translation of diagnostic reasoning into concrete actions. As shown in Section 3, this process retains the same two levels—life-threatening risk and patient-expressed problems—while expanding into multiple dimensions, such as time management, communication, emotional regulation, fostering comfort, and supporting autonomy.

These findings reinforce the multidimensional nature of intervention, aligning with Simmons and Alfaro-LeFevre [3,7], who describe clinical reasoning as an integration of cognitive, technical, relational, and emotional competencies.

Participants’ descriptions of empathic communication, confidence, and personalization confirm the central role of the therapeutic relationship in shaping clinical reasoning, fully consistent with the excerpts analyzed in Section 3.

### 4.3. Clinical Reasoning as a Relational and Context-Dependent Process

As highlighted in the conceptual model (Figure 3), clinical reasoning is not an isolated cognitive event but a relational and contextual process shaped by interactions of nurses, patients, the latter’s family, and the organizational structure of the emergency department.

This finding is consistent with Peplau’s interpersonal theory and aligns with contemporary perspectives describing reasoning as an intersubjective and collaborative process [31,32,33,34].

### 4.4. Influence of Emergency Department Sectors on Reasoning

The results also demonstrated that reasoning varies across emergency department sectors. In first-contact areas such as triage, reasoning is rapid and oriented toward immediate classification, whereas in second-contact areas, it becomes more reflective, iterative, and dependent on continuous reassessment.

This sector-dependent variation supports Benner’s concept of situated knowledge and is consistent with research showing the influence of workload, resources, and team dynamics on clinical reasoning [15,16,17].

### 4.5. Integration and Contribution of the Conceptual Model

The conceptual model developed in this study integrates the two subcategories—Diagnostic Nursing Assessment and Therapeutic Nursing Intervention—into a spiral structure of perception, interpretation, decision, and action. This model visually and conceptually synthesizes the dynamic, adaptive, and cyclical nature of reasoning observed in the narratives.

The model also highlights the role of operational axes (risk management, information and time management, emotional regulation, comfort, and autonomy) and contextual layers (therapeutic relationship stages and department sectors) in shaping how reasoning unfolds, directly reflecting the multilayered findings presented in Section 3.

### 4.6. Contribution to Knowledge

This study provides an in-depth explanation of how nurses construct their clinical reasoning while managing critically ill patients in an emergency department. The integration of assessment, intervention, relational engagement, and contextual awareness provides a comprehensive and empirically grounded contribution to the theoretical understanding of clinical reasoning in emergency care.

Effective case management, anticipation of complications, and continuous situational awareness emerge as pillars of safe practice, reinforcing the essential role of systematic assessment, vigilant observation, and high-quality therapeutic relationships in supporting evidence-based decision making.

### 4.7. Implications for Future Research

Although this study has deepened the understanding of clinical reasoning in emergency nursing, it also opens new avenues for future investigation. It would be valuable to extend the analysis to other practice contexts, such as primary healthcare, long-term care, and community health, where decision-making dynamics may take on different characteristics.

Longitudinal and multicenter studies could further explore how clinical reasoning evolves throughout a nurse’s professional career, clarifying the influence of experience and advanced education. Moreover, integrating mixed methods designs combining qualitative and quantitative data could strengthen the robustness of findings and enable correlations among clinical reasoning, health outcomes, and patient safety indicators.

Finally, it would be relevant to examine the impact of innovative pedagogical strategies, such as high-fidelity simulation, digital technologies, and virtual learning environments, on the development of clinical reasoning skills. Such research could consolidate a growing body of knowledge that reinforces evidence-based nursing practice and promotes excellence in professional performance across complex healthcare settings.

### 4.8. Implications for Patients

For patients and families, the implications translate directly into the safety and quality of care. Studies have shown that nurses with more highly developed clinical reasoning skills reduce the occurrence of errors and adverse events, thereby improving health outcomes. The present study confirms that robust clinical reasoning enables nurses to anticipate complications, prevent risks, and tailor interventions, ensuring a faster, more effective, and more humanized response.

## 5. Limitations

This study had some limitations. Although participant recruitment was facilitated by the nurse manager, there was no evidence of administrative restriction, and both the institution and the participating nurses demonstrated full commitment to supporting our research. However, the high workload in the emergency department occasionally limited the time available for conducting interviews, requiring flexibility in scheduling. Additionally, this study was carried out in a single emergency department, which may restrict the contextual variability captured. Replicating this study in other emergency settings would be valuable to explore how organizational structures, workflows, and team cultures influence nurses’ clinical reasoning. Finally, although theoretical sampling guided recruitment, the perspectives obtained reflect the particular characteristics of this context and may not be directly transferable to other institutions.

## 6. Conclusions

This study provided an in-depth understanding of the Nursing Clinical Reasoning Process in emergency settings, revealing it as a dynamic, continuous, and relational phenomenon composed of two central dimensions: Diagnostic Nursing Assessment and Nursing Therapeutic Intervention. These interdependent subprocesses confirm that clinical reasoning constitutes the core of professional nursing competence, underpinning decision making and the quality of care delivery.

The findings demonstrate that clinical reasoning is not limited to a technical or cognitive process but is deeply rooted in the therapeutic relationship established between the nurse and the patient and the latter’s family. In other words, nursing care is intrinsically linked to interpersonal interaction and the development of a relationship of trust, commitment, and negotiation, all of which are essential elements for effective clinical decision making and for the provision of meaningful and humanized care.

Another significant conclusion concerns the influence of context. First-contact areas (such as triage and admission) demand speed and prioritization skills, whereas second-contact areas (observation and follow-up) allow for deeper and more continuous assessments. This plasticity of clinical reasoning in response to environmental demands underscores that nursing phenomena are constructed through the interaction between individuals and their context.

From a clinical practice perspective, this study confirms that developing robust clinical reasoning is crucial to care quality and patient safety, as it enables nurses to anticipate complications, prevent risks, and ensure timely and appropriate interventions. Therefore, it can be concluded that investing in the education and training of clinical reasoning in nursing is essential, both in undergraduate education and in continuing professional development. The use of methodologies such as clinical simulation, case-based learning, and structured reflection can enhance nurses’ ability to make sound, evidence-informed decisions, thereby improving health outcomes and promoting person-centered care.

In summary, this study contributes to the development of a theoretical explanatory model of the Nursing Clinical Reasoning Process in emergency contexts, which can serve as a foundation for designing educational, training, and management strategies in nursing. Such a model supports evidence-based practice and strengthens patient safety in high-complexity clinical environments, particularly in the care of critically ill patients.

## Data Availability

The data presented in this study are openly available in Reitoria (REIT) at http://hdl.handle.net/10451/49995 (accessed on 15 October 2025).

## Abbreviations

The following abbreviations are used in this manuscript:

BSN	Bachelor of Science in Nursing
CE	Ethics Committee
CH	Hospital Center
GT	Grounded Theory
MSN	Master of Science in Nursing
